# Exploring autophagy-related prognostic genes of Alzheimer’s disease based on pathway crosstalk analysis

**DOI:** 10.17305/bjbms.2021.7019

**Published:** 2022-04-02

**Authors:** Fang Qian, Wei Kong, Shuaiqun Wang

**Affiliations:** Department of College of Information Engineering, Shanghai Maritime University, Shanghai, China

**Keywords:** Alzheimer’s disease, autophagy, prognostic signature, pathway crosstalk, weighted gene coexpression network analysis, pathway analysis method based on global influence

## Abstract

Recent studies have shown that different signaling pathways are involved in the pathogenesis of Alzheimer’s disease (AD), with complex molecular connections existing between these pathways. Autophagy is crucial for the degradation and production of pathogenic proteins in AD, and it shows link with other AD-related pathways. However, the current methods for identifying potential therapeutic targets for AD are primarily based on single-gene analysis or a single signal pathway, both of which are somewhat limited. Finding other methods are necessary for providing novel underlying AD therapeutic targets. Therefore, given the central role of autophagy in AD and its interplay with its pathways, we aimed to identify prognostic genes related to autophagy within and between these pathways based on pathway crosstalk analysis. The method of pathway analysis based on global influence was applied to find the feature mRNAs involved in the crosstalk between autophagy and other AD-related pathways. Subsequently, the weighted gene coexpression network analysis was used to construct a coexpression module of feature mRNAs and differential long non-coding RNAs. Finally, based on two autophagy-related crosstalk genes (CD40 and SMAD7), we constructed a prognosis model by multivariate Cox regression, which could predict the overall survival of AD patients with medium-to-high accuracy. In conclusion, we provided an effective method for extracting autophagy-related significant genes based on pathway crosstalk in AD. We found the biomarkers valuable to the AD prognosis, which may also play an essential role in the development and treatment of AD.

## INTRODUCTION

Alzheimer’s disease (AD) is one of the common forms of dementia, and its typical pathological features are the presence of amyloid (Aβ) deposition, hyperphosphorylated tau protein aggregation, and neurofibrillary tangles in the brain [1]. Although the pathological relevance is known, the exact pathogenesis of AD is still poorly understood. Research in recent years has discovered and explained many signal pathways related to AD, among which the key role of the autophagy pathway in AD is becoming more and more prominent [2]. Autophagy is a crucial regulator of the Aβ and tau proteins production. Aβ and tau proteins can induce autophagy as well to promote its clearance through the mTOR pathway or independently [3,4]. Many studies have shown that normal autophagy protects neurons, but dysfunctional autophagy may increase the deterioration of neurons in AD [5,6]. In addition, there are interactions between autophagy and other related signaling pathways, which provide new possibilities for exploring the pathogenesis and therapeutic targets of AD.

With the innovation of RNA sequencing technology and bioinformatics analysis, the identification of hub genes and functional pathways in AD has developed rapidly [7]. In addition to mRNA, long non-coding RNA (lncRNA) has also been found to be involved in the pathological progress of AD, including the induction of autophagy to promote the clearance of Aβ or tau protein, the inhibition of neuroinflammation, and other biological processes [8,9]. Studying the molecular mechanism of these RNAs and their interaction with autophagy will provide promising AD diagnosis and treatment methods [10,11].

Although these RNAs play a crucial role in AD, most of the current methods for their excavation are based on bioinformatics analysis and competition with endogenous RNA hypotheses, which may ignore their interconnection with autophagy or crosstalk between pathways. In excavating disease-causing genes, the above methods may fail to identify more meaningful molecular targets. Therefore, it is necessary to find new effective strategies.

With the accumulation of high-throughput genome-wide expression data, researchers can systematically study the functional relationship of single or several genes in diseases [12]. It is well known that genes do not function in isolation but work together within various metabolic, regulatory and signaling pathways. Furthermore, increasing evidence shows that pathway-based methods are generally superior to gene-based counterparts [13].

Methods based on pathway analysis can explain complex biological processes and biological significance. AD is a multifactorial disease involving multiple cell signaling pathways, so crosstalk within and between pathways exists. Because the number and combinations of signals are limited, crosstalk between pathways can create novel input/output combinations. Having more input/output combinations increases the possible ways of the signaling information flow within the cell, allowing more diverse phenotypes. Thus, genes generated by crosstalk play an essential role in the generation and development of disease [2,14,15].

Several recent techniques have used path topology information to identify dysfunctional paths. The pathway analysis based on the global influence (PAGI) algorithm utilizes gene networks [16], looking for dysregulated pathways in diseases by considering the internal effects of pathways and crosstalk between pathways, which provides the possibility for the AD exploration.

Studies have shown that lncRNA can directly mediate crosstalk in pathways by cooperating with coding genes that play an important role in diseases [17]. In addition, lncRNAs and mRNAs can compete with each other through miRNAs response elements to regulate AD progress [18]. Based on the interaction of autophagy with the RNA mentioned above and other signal pathways in AD, it is believed to be a novel idea to investigate the role of autophagy-related genes in AD and their pathogenic mechanisms in diseases from the perspective of pathway crosstalk.

Our research introduced a novel method to identify prognostic genes related to autophagy in AD. First, the PAGI pathway analysis algorithm was applied to gene expression data in order to obtain pathways and crosstalk genes associated with AD. Second, the WGCNA algorithm was used to select significant genes with coexpression relationships, and genes in the same expression module may have similar biological functions [19].

Finally, the survival analysis was applied to significant genes, and the prognostic genes of AD were extracted. Results showed that based on PAGI, more than 36 autophagy-related pathways dysregulated and crosstalk with each other in AD. Furthermore, 103 lncRNAs and 650 mRNAs related to autophagy with coexpression relationships were identified using WGCNA analysis. Next, CD40 and SMAD7 were identified as prognostic genes of AD, which was also verified in the external AD dataset. Finally, we performed prognostic survival analysis and differential expression analysis on the other AD data to verify the prognostic genes. The experimental results confirmed our conclusions.

## MATERIALS AND METHODS

### Data source

The data used in this study was obtained from subjects of the Religious Order Study (ROS) or the Rush Memory and Aging Project (MAP), which are two prospective clinical-pathological cohort studies of aging and dementia. The two studies (collectively referred to as ROSMAP) share clinical and neuropathological standards, allowing joint data analysis [20,21]. Moreover, the ROSMAP study is stored in the AD Knowledge Portal (https://adknowledgeportal.synapse.org/).

The gene expression profile (syn8691134) and clinical information needed for this research were obtained from the AD Knowledge Portal database (the raw count data can be obtained online at https://www.synapse.org/#!Synapse: syn8691134, the filtered raw count data can be obtained online at https://www.synapse.org/#!Synapse: syn8456637, and the clinical data were downloaded online at https://www.synapse.org/#!Synapse: syn3191087). According to clinical information, we screened 155 AD samples and 86 normal samples as the data for this study. The validation dataset (syn4009614) was obtained from the AMP-AD Knowledge Portal database (the normalized data can be obtained online at https://www.synapse.org/#!Synapse: syn4009614).

**FIGURE 1:**
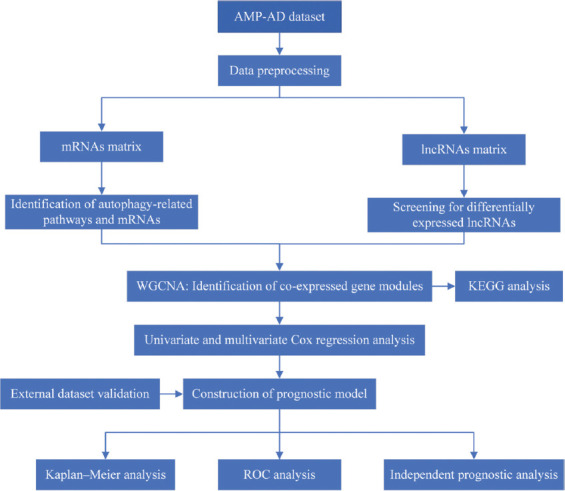
Framework of the experiment.

We downloaded the autophagy gene file from the Human Autophagy Database (http://www.autophagy.lu/) and HAMdb (http://hamdb.scbdd.com/) for the annotation of autophagy genes on the pathway. The experimental flowchart of this paper is shown in the Figure 1, and we will discuss each part in detail later.

### Data preprocessing

According to the human genome assembly GRCh38, the ensemble ID of the raw count data was switched to the gene symbol. According to the gene type annotation, the genes in the raw data were divided into 19,677 mRNAs and 14,259 lncRNAs. We only kept the highly expressed mRNA (obtained from filtered count data syn8456637, deleted genes whose count is <1 CPM in at least 50% of the samples, and deleted genes whose length and GC content are missing). Then, we performed TPM standardization and normalization on the filtered mRNA expression profile and used the SVA package to eliminate batch effects (sequencing batch of samples, nine batches in total) in the standardized data.

The R package “DESeq2” was used to identify differentially expressed lncRNAs (DElncRNAs) [22]. First, we used the DESeqDataSetFromMatrix function to convert the count matrix into a DESeqDataSet (DDS) object. The formula of the design parameter of DESeqDataSetFromMatrix function is as follows: design=~batch+group, where batch represents the sequencing batch information of the sample (batch value is from 0 to 8), group is the grouping information of the sample (the samples were divided into diseased group and normal group). Then, we used the DESeq function to normalize (by calculating the size factor of each column of samples in the count matrix) DDS and analyze the differential expression of lncRNAs in normal and diseased samples. The selected criteria for screening differentially expressed lncRNAs was taking *p* < 0.05 into consideration, and a log2FC range of –0.5-0.5 was rejected.

### PAGI

The PAGI algorithm is a pathway analysis method based on global influence, which identifies dysregulated pathways by considering both within-pathway effects and crosstalk between pathways. We used the PAGI algorithm to identify dysregulated pathways in AD. Principle of the PAGI algorithm is as follows:

First, constructing the global gene-gene network. Based on the structural information and genetic relationship of each pathway in KEGG, the “iSubpathwayMiner” system was used to construct a global gene network that reflected the relationship between and within a pathway [23,24].

Second, calculating the global dysregulated score (GDS). GDS was used to assess the degree to which genes are affected by the internal effects of pathways and crosstalk between pathways. The Random Walk with Restart (RWR) algorithm captures global relationships within a network and can calculate the node’s proximity to a set of source nodes [25]. In the application process, the two-sample (diseased and normal samples) t-test was performed to evaluate the extent of differential expression (t-score). All genes represented in the gene expression profile were mapped to the global network as source nodes. The RWR algorithm was modified by combining the t-score and the global network topology to calculate GDS and reflect the global influence of the gene on the source nodes. The revised algorithm formula is as follows:


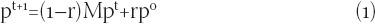


Where, M is the column-normalized adjacency matrix of the global network graph G, P^t^=(P_1_^t^,P_2_^t^,...P_n_^t^) is the node vector at time t, and its i^th^ element P_1_^t^ represents the probability of being at node i at time t, and n represents the number of all nodes (genes) in G. r is the restart probability, which controls the degree to which the random traverser returns to the source node in each iteration.

The initial probability P^0^=(P^0^_1_,P^0^_2_,...P^0^_n_) is normalized to the unit vector 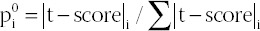. The higher the P^o^_i_ of gene i, the greater the degree of disturbance to other genes. p^t^ can reach a stable state p^∞^ after multiple iterations, it was used to measure the GDS of genes. The GDS of gene i was assigned by the normalized P^∞^_i_ as: GDS_i_=(P^∞^_i_−min(P^∞^))/(max(P^∞^)−min(P^∞^)). Through this method, the GDS of each gene in the global network can be obtained.

Third, identification of dysregulated pathways. The gene list L={g_1_,g_2_,g_3_,…,g_n_} consists of all genes in the expression profile sorted according to t_j_^l+GDS^_j_, t_j_ represents |t-score| of gene j, and GDS_j_ represents GDS of gene j. The dysfunction score of p path is calculated based on the information of its gene mapping in the L path and is calculated by cumulative distribution functions (CDFs). The CDFs of Inp (genes in P) and Notp (genes in L, not in P) are used to evaluate the fraction of genes in p weighted by their correlation (t_j_^l+GDS^_j_), and the fraction of genes not in P presents up to a given position i in L. The formula is as follows:


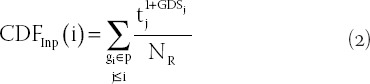


and


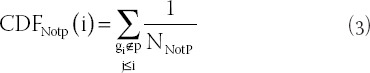


Where,
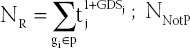
 represents the number of genes in L not in P. With the position I walking down the list L, the formula for calculating the dysfunction score of path P is as follows:


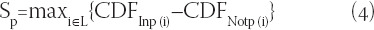


Finally, the significantly dysregulated pathways in AD obtained according to the false discovery rate (FDR<0.01) were used as candidate pathways. According to the autophagy gene annotation results of each candidate pathway and the literature review, the pathways that interact with autophagy were selected as the feature pathways, and the mRNAs in the feature pathway were used as the feature mRNAs.

### Construction of coexpression gene modules based on WGCNA analysis

The R package “WGCNA” was used to identify DElncRNAs and feature mRNAs with coexpression relationship. Therefore, the feature mRNAs and DElncRNAs served as the input of WGCNA. First, the absolute value of the Pearson correlation coefficient between genes was used to construct the correlation matrix (S_i,j_, i and j indicate the i^th^ and j^th^ gene). The threshold of the fitting index was set to 0.85 (R^2^>0.85) to fit with the scale-free network. When the fitting index reached 0.85, the β value (soft threshold) that maximized the average connectivity was selected to perform a power-law operation to convert the correlation matrix into an adjacency matrix (a_i,j_, a_i,j_=|S_i,j_|^β^) [26].

Then, the pickSoftThreshold function was used to calculate the corresponding and average connectivity for different β values (the β values were set between 1 and 20). Next, we transformed the adjacency matrix into a topological overlap matrix. We used the DynamicTreeCut algorithm to construct the average linkage hierarchical clustering dendrogram [27]. Finally, we calculated the module Eigengenes, hierarchically clustered the modules, and merged similar modules [28].

### Functional enrichment analysis

KEGG analysis was used to explore the biological functions of genes on coexpressed modules [29]. The filter condition of the enrichment pathway was that *p*-value and the adjusted *p*-value (q-value) were both less than 0.05. Finally, according to the results of KEGG enrichment, the gene modules needed in this study were obtained, and the genes in the modules were used as AD significant genes.

### Cox proportional hazards regression analysis

To obtain significant genes related to the prognosis of AD, we used the R package “survival” to perform univariate and multivariate Cox regression analysis on the AD candidate biomarkers (genes in the turquoise module). To verify the validity of the prediction model, we used syn8691134 as the training dataset, and syn8691134 and syn4009614 datasets were combined as the testing dataset. First, we extracted the expression data of genes belonging to the turquoise module in the training dataset and the testing dataset, and the expression data of their overlapping genes were used as the input of univariate Cox regression analysis.

Then, univariate Cox regression analysis was used to identify genes significantly related to the overall survival (OS) of patients with AD. The criterion for screening genes related to the OS of patients with AD is that *p* < 0.01 was considered. Next, we performed multivariate Cox regression analysis on the genes screened by univariate Cox regression analysis and constructed a prognostic-related model of AD. We used the stepwise selection of variables based on the lowest Akaike information criterion (AIC) to optimize the prognostic-related model of AD. We calculated the risk score of each patient, which can be used to divide AD patients into a high-risk group and a low-risk group. The formula is as follows:


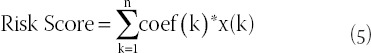


Where, coef(k) represents the Cox regression coefficient, x(k) represents the expressive value of each genes, and n represents the number of genes.

Finally, the Kaplan–Meier (KM) curve and forest plot of multivariate Cox regression analysis were generated by R package “survival.” The KM curve was used to judge whether it exists a difference in survival between the high-risk and the low-risk groups. The forest plot was used to judge whether the risk score is an independent prognostic factor affecting OS. Then time-dependent receiver operating characteristic (ROC) curve and the multi-index ROC curve were used to evaluate the accuracy of the prognostic-related model by R package “timeROC.”

### Statistical analysis

According to the test condition, Wilcoxon test was used to perform statistical comparisons between two groups of data, and *p*< 0.05 was used to indicate statistical significance. The GSE118553 and GSE5281 AD dataset were obtained from the GEO platform (https://www.ncbi.nlm.nih.gov/geo/).

## RESULTS

### Data preprocessing results and DElncRNAs

After quality control and TPM standardization of mRNAs data, 13,556 mRNAs were obtained. Then, the standardized data were adjusted in batches (nine batches in total) through the combat function of the SVA package, and the adjusted data were reserved as the input of the PAGI algorithm.

To identify differentially expressed lncRNAs, we used the DESeq2 package to standardize the gene expression profile of lncRNAs and analyze differential expression. The lncRNAs with *p* < 0.05 and |log2FC|>0.5 were extracted as statistically significant differential genes. Finally, 180 differentially expressed lncRNAs were obtained, of which 75 were upregulated and 105 were downregulated. The top 30 differential genes (15 down and 15 up) are shown in the Supplemental Table 1.

### Exploring autophagy-related pathways based on PAGI

To explore autophagy-related biomarkers in AD from the perspective of pathway crosstalk, we applied the mRNA expression profile of AD to the PAGI algorithm. According to the FDR value <0.01, 94 pathways related to AD were screened out. Then, by consulting the literature and annotating the autophagy genes on each pathway, 36 autophagy-related pathways and crosstalk genes required for this study were screened out. A total of 1436 crosstalk genes (feature mRNAs) were included in the 36 pathways, which were one of the input data of the WGCNA algorithm.

**FIGURE 2:**
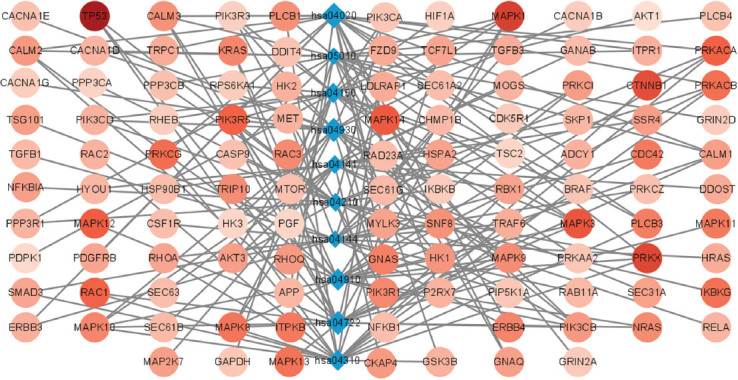
Network connection diagram of 10 pathways and their crosstalk genes. The blue diamond nodes represent different pathways, and the size of the nodes represents the score of the pathway in PAGI. The circular node connected to the blue diamond node represents the genes included in the pathways. The shade of the color represents the level of the gene’s GDS score. The darker the node’s color, the higher the GDS score represents the gene. hsa04141: Protein processing in endoplasmic reticulum. hsa04210: Apoptosis. hsa05010: Alzheimer’s disease. hsa04150: mTOR signaling pathway. hsa04910: Insulin signaling pathway. hsa04722: Neurotrophin signaling pathway. hsa04144: Endocytosis. hsa04930: Type II diabetes mellitus. hsa04020: Calcium signaling.

Ten out of the 36 pathways were confirmed to be related to autophagy in AD and are shown in Table 1, and information about the remaining pathways is shown in Supplemental Tables 2 and 3. The 10 pathways and their crosstalk genes (GDS score ranks top 20 in the pathway) were displayed in a pathways-gene network in Figure 2. From Figure 2, it can be seen that genes in hsa047229 (Neurotrophin signaling pathway) and hsa04310 (Wnt signaling pathway) pathways had higher GDS scores, while genes on hsa04141 (Protein processing in endoplasmic reticulum) pathways had lower GDS scores.

**TABLE 1:**
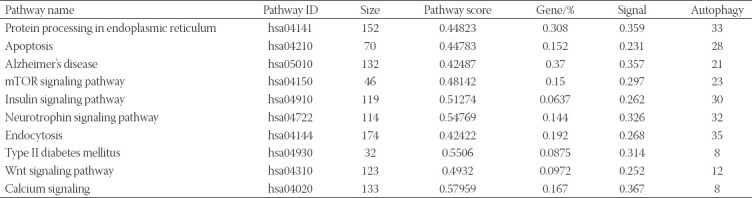
Ten pathways related to autophagy identified by the PAGI

**SUPPLEMENTAL FIGURE 1:**
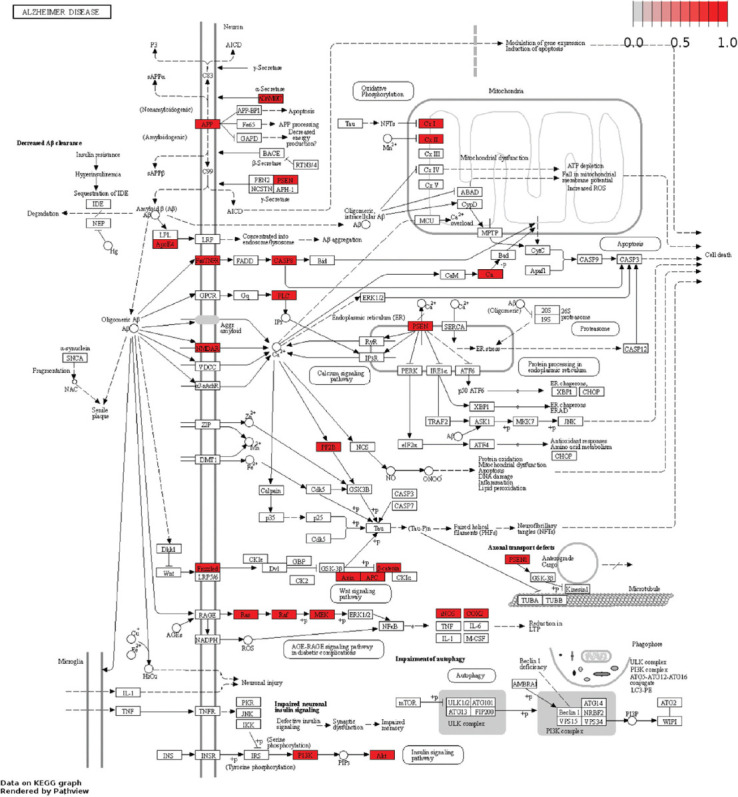
Genes associated with other diseases act to Alzheimer’s disease pathway. The red rectangles represent biological processes involved in genes associated with other diseases.

In addition, based on the KEGG database (https://www.kegg.jp/), we explored other diseases associated with autophagy-related crosstalk genes in AD (Supplemental Table 4) and mapped related genes in AD pathways (Supplemental Figure 1). These genes, which are related to other diseases, may affect the progression of AD by participating in signaling pathways such as the calcium signaling pathway, insulin signaling pathway and calcium signaling pathway in AD.

In Table 1, the first column is the pathway name based on PAGI algorithm screening, the second column is the pathway ID, and the third column “Size” indicates the number of genes contained in the pathway, the fourth column “Pathway Score” is the score after the pathway passes the PAGI algorithm, the fifth column “Gene\%” is the percentage in the gene list before running enrichment peak, and the sixth column “Signal” indicates the intensity of the enrichment signal, and the seventh column is the number of autophagy genes included in the pathway.

It can be seen from Table 1 that the scores of these 10 pathways were all higher than 0.4, and the pathway score of AD (hsa05010) pathway was 0.42487, which directly proved the effectiveness of the pathway selection through PAGI. Furthermore, the pathway score of the mTOR signaling pathway (hsa04150) was 0.48142, which is currently one of the most promising targets for autophagy-related AD therapy [3]. Moreover, autophagy genes (489 in total) in the selected pathways all account for a high proportion, which provided a basis for extracting autophagy-related crosstalk genes.

### Analysis of autophagy-related coexpression module

**FIGURE 3:**
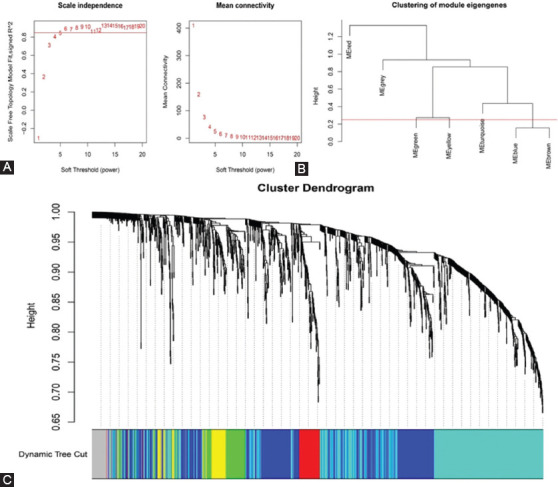
Network construction of coexpressed genes. (A) Analysis of the scale independence and mean connectivity for various soft-threshold powers; (B) the cluster dendrogram of module Eigengenes; (C) dendrogram clustered based on a dissimilarity measure (1-TOM).

To obtain feature mRNAs (autophagy-related crosstalk genes) and lncRNAs with a coexpression relationship, we used the WGCNA algorithm to construct a coexpression module for it. First, we obtained the count matrix composed of differentially expressed lncRNAs and autophagy-related crosstalk genes. β=6 (R^2^=0.89) was set as the soft-thresholding parameter to construct the scale-free network (Figure 3A). The number of genes in each module was defined as at least 50. Next, seven modules were identified based on DynamicTreeCut algorithm. Finally, based on the module Eigengenes, the height of cut was 0.25 to merge similar modules (Figure 3B). Moreover, six coexpression modules were identified (Figure 3C). The six modules are shown in Table 2. From Table 2, we can see that the number of autophagy genes in the turquoise module is the largest among the six modules.

**TABLE 2:**
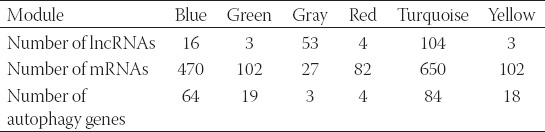
Six coexpressed gene modules obtained by WGCNA

**FIGURE 4:**
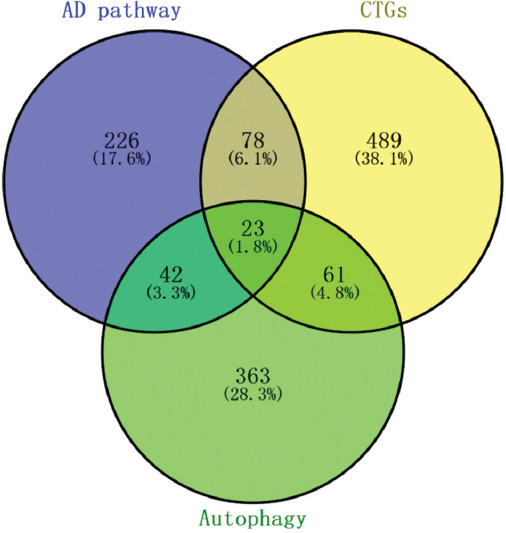
Venn diagram showing the overlap of crosstalk genes, autophagy-related genes (autophagy), and Alzheimer’s disease pathway genes (AD pathway, hsa05010).

KEGG pathway enrichment analysis was performed on the genes of each module, and the turquoise module was finally determined as the research object according to the analysis results. The turquoise module includes 103 lncRNAs and 650 mRNAs. One hundred and one of the 650 mRNAs are located in the AD pathway (369 genes), and 45 are autophagy genes (489 genes), as shown in Figure 4. From Figure 4, the crosstalk genes in the turquoise module overlap more with the genes in autophagy and AD pathway (hsa05010), indicating that the coexpression module we selected has a correlation with AD and autophagy. Pathway enrichment analysis of gene modules was implemented by David (https://david.ncifcrf.gov/) [30].

**FIGURE 5:**
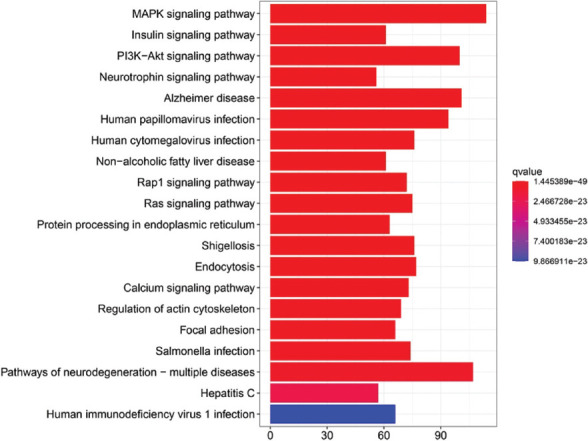
The results of pathway enrichment. The horizontal axis is the number of genes in the pathway, and the vertical axis is the pathway list. Red to blue indicates the q-value (adjusted p-value).

The pathway enrichment results of the turquoise module are shown in Figure 5. David obtained the first 20 pathways with p < 0.05, including AD pathway, pathways of neurodegeneration-multiple diseases, and PI3K-Akt signaling pathway [31]. The discovery of the above pathways directly proved the significance of the turquoise module as a research object. Moreover, the genes on the turquoise module are also involved in MAPK signaling pathway [32], protein processing in endoplasmic reticulum [33], calcium signaling pathway [34], focal adhesion [35], insulin signaling pathway [36], neurotrophin signaling pathway [37], regulation of actin cytoskeleton [38], endocytosis [39], and Ras signaling pathway [40].

In Table 2, the first row is the name of the six coexpression modules, and each column represents the number of lncRNA, mRNA, and autophagy genes contained in the module.

### Construction of an autophagy-related prognostic gene model

The clinical data downloaded from the AMP-AD database were sorted and screened. Then, the survival time and clinical characteristics (braaksc: Braak stage, ceradsc: Assessment of neuritic plaques, and dcfdx_lv: Clinical cognitive diagnosis summary at last visit) of 82 AD patients in the training dataset and 137 AD patients in the testing dataset were obtained (Supplemental Table 5). Based on the results of WGCNA and KEGG, we selected the genes in the turquoise module (754 genes) for the following analysis. Among the overlapping genes of training dataset and testing dataset, 631 genes belong to the turquoise module. Then, we used the expression data of these 631 genes in training dataset as the input of univariate Cox regression analysis. According to *p*-value (*p* < 0.01), we screened 12 genes that were significantly related to the prognosis of AD (Table 3). Subsequently, we performed multivariate Cox analysis on 12 prognostic-related genes obtained from univariate Cox regression analysis. Afterward, according to the lowest AIC value, the prognostic risk model of two genes (CD40 and SMAD7) of the training dataset was constructed. The risk score is expressed as: riskScore = (CD40 exp.* –1.13) + (SMAD7 exp.* –1.41) (Table 4). Patients with AD in training dataset and testing dataset were divided into low- and high-risk groups based on the median risk score in the training dataset.

**TABLE 3:**
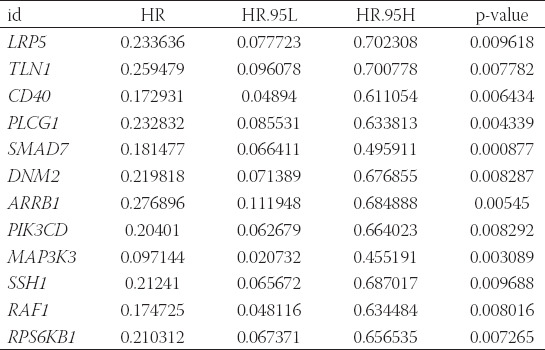
Twelve autophagy-related crosstalk genes associated with AD overall survival time were obtained from univariate Cox regression analysis

**TABLE 4:**
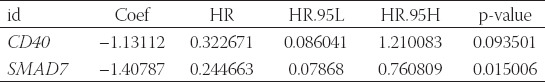
Two autophagy-related prognostic genes were obtained from multivariate Cox regression analysis

**FIGURE 6:**
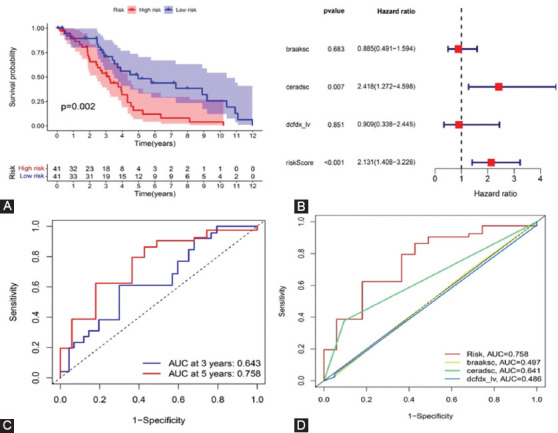
Prognostic significance analysis of the Alzheimer’s disease training dataset. (A) Kaplan–Meier curve to compare OS of high risk with low-risk samples (*p* = 0.002); (B) forest plot of multivariate independent prognostic analysis. The square on the horizontal line shows the hazard ratio (HR), and the horizontal line represents the 95% confidence interval; (C) time-dependent receiver operating characteristic (ROC) curve analysis of the risk score model for predicting 3- and 5-year OS; and (D) multi-index ROC curve. The curve area is used to assess the accuracy of the risk model (model AUC = 0.758).

According to the median risk score, 82 AD patients in the training dataset were divided into high-risk (n = 41) and low-risk (n = 41) groups. It can be seen from the KM curve that the OS rate of high-risk patients was significantly lower compared with low-risk patients within 5 years (Figure 6A). Multivariate Cox regression analysis revealed that the risk score of prognostic risk model (*p* = 0.002) was an independent prognostic factor affecting the OS of patients with AD in training dataset (Figure 6B). The area under the ROC curve (AUC) was calculated to assess the predictive ability of the model. The 3- and 5-year AUCs were 0.643 and 0.758 (Figure 6C). The multi-index ROC curve showed that the AUC value of the risk score based on the prognostic risk model was greater than 0.7 (AUC = 0.758), which was more significant than other clinical prognostic indicators, such as braaksc, ceradsc, and dcfdx_lv (Figure 6D).

**FIGURE 7:**
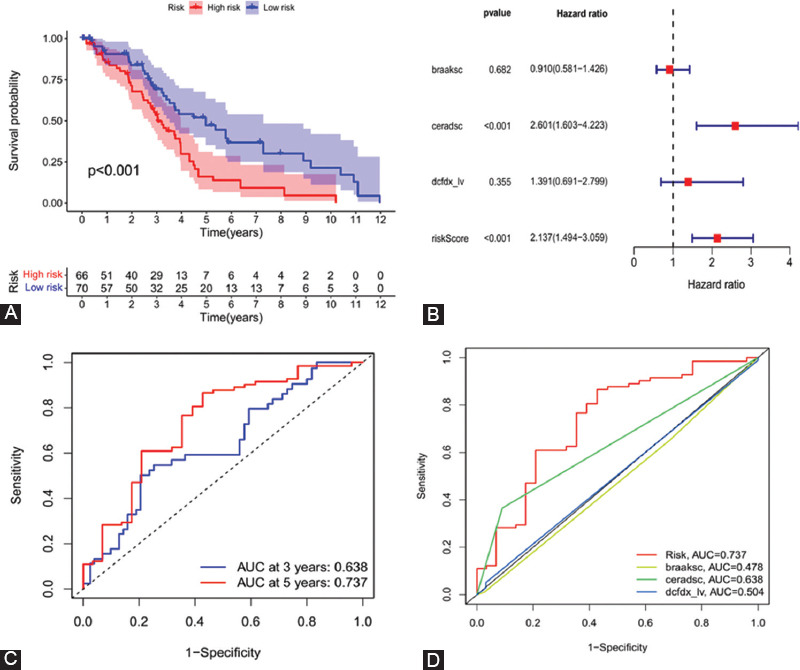
Prognostic significance analysis of the Alzheimer’s disease testing dataset. (A) Kaplan–Meier curve to compare OS of high risk with low-risk samples (*p* < 0.001); (B) forest plot of multivariate independent prognostic analysis. The square on the horizontal line shows the hazard ratio (HR), and the horizontal line represents the 95% confidence interval; (C) time-dependent receiver operating characteristic (ROC) curve analysis of the risk score model for predicting 3- and 5-year OS; and (D) multi-index ROC curve. The curve area is used to assess the accuracy of the risk model (model AUC = 0.737).

The results of the Kaplan–Meier analysis showed that patients with high-risk scores had less survival time in the testing datasets (*p* < 0.001) (Figure 7A). The forest plots of the multivariate independent prognostic analysis indicated that the risk score of prognostic risk model (*p* < 0.001) was an independent prognostic factor affecting the OS of AD patients in testing dataset (Figure 7A and B). From time-dependent ROC curve of testing dataset, the 3- and 5-year AUCs were 0.672 and 0.746 (Figure 7C). The multi-index ROC curve showed that the AUC value of the risk score based on the prognostic risk model was >0.7 (AUC = 0.737), which was more significant than other clinical prognostic indicators, such as braaksc, ceradsc, and dcfdx_lv (Figure 7D). The above results proved the accuracy of the prognostic risk model.

### The expression of CD40 and SMAD7

**FIGURE 8:**
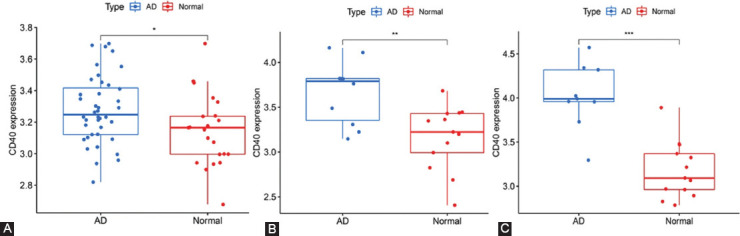
Relative expression levels of CD40 in Alzheimer’s disease and control sample (“*” represents *p* < 0.05, “**” represents *p* < 0.01, “***” represents *p* < 0.001). (A) frontal cortex of GSE118553; (B) hippocampus of GSE5281; and (C) posterior cingulate of GSE5281.

**FIGURE 9:**
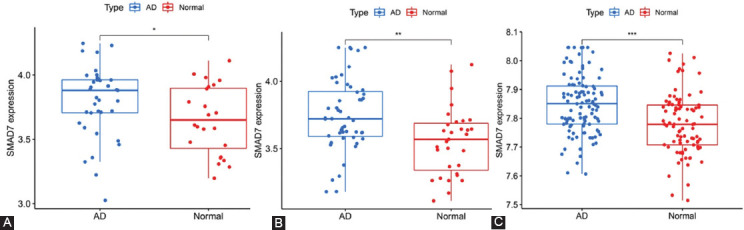
Relative expression levels of SMAD7 in Alzheimer’s disease and control sample (“*” represents *p* < 0.05, “**” represents *p* < 0.01, “***” represents *p* < 0.001). (A) Entorhinal cortex of GSE118553; (B) temporal cortex of GSE118553; and (C) Syn4009614.

Finally, we explored the expression of CD40 and SMAD7 in AD and normal brain tissues using GSE5281, GSE118553, and syn4009614. As shown in the following figures below, we observed that CD40 (Figure 8A-C) and SMAD7 (Figure 9A-C) were significantly upregulated in AD.

## DISCUSSION

### Extract autophagy-related pathways based on pathway crosstalk

A variety of signal pathways in AD are involved in the generation and development of disease, and crosstalk is inevitable between these signal pathways. Crosstalk between pathways provides a novel combination of non-linear response dysfunction. Furthermore, genes generated by crosstalk play an essential role in the generation and development of the disease. As the primary regulator of the production and clearance of Aβ and tau protein in AD, it has recently been discovered that the effect of autophagy on AD is related to its interaction with various signaling pathways and known AD biomarkers [2,8]. Therefore, exploring the autophagy-related biomarkers in AD from the perspective of pathway crosstalk has become the direction of our exploration.

First, we obtained the pathways with crosstalk in AD as well as the crosstalk genes on the pathways by the PAGI algorithm. We obtained 96 pathways with crosstalk, which further confirmed the extensive crosstalk between signaling pathways in AD. Then, by consulting the literature and annotating the autophagy genes of each pathway, 36 pathways were identified as autophagy-related crosstalk pathways. The genes on the pathways were identified as autophagy-related crosstalk genes and were reserved for further analysis. The results showed that autophagy-related crosstalk pathways in AD were mainly involved in the AD signaling pathway, mTOR signaling pathway, calcium signaling pathway, Wnt signaling pathway, apoptosis, insulin signaling pathway, neurotrophin signaling pathway, Type II diabetes mellitus, and other pathways.

These signaling pathways are closely related to the progression of AD. The mTOR signaling pathway is a crucial regulator of autophagy in AD. Previous studies have found that increasing mammalian target of mTOR signaling raises tau levels and phosphorylation [41]. Intracellular calcium signaling (Ca^2+^) pathway dysregulation is centrally involved in AD pathogenesis (the aggregation of pathogenic Aβ, synapse loss and dysfunction and phosphorylation of tau) [34]. Furthermore, our results found that the calcium signaling pathway had a higher crosstalk score (pathway score = 0.57959). Multiple signaling pathways have been reported to play an important role in AD by regulating Ca^2+^ homeostasis, including Wnt signaling pathway, neurotrophin signaling pathway and apoptosis [2].

The above results revealed that Ca^2+^ might be involved in the occurrence and progression of AD through extensive crosstalk with other pathways. Insulin is a crucial factor regulating cell growth, autophagy, synaptic plasticity and cognitive function. Dysregulation of insulin signaling can cause neurofibrillary tangles and Aβ plaques (the main pathological features of AD) [36]. The above results indicate that the pathways we screened and the crosstalk genes on the pathways play a crucial role in the AD occurrence, as well as pathological progression.

We also performed functional enrichment analysis of autophagy-related crosstalk genes obtained by WGCNA, which revealed that these autophagy-related crosstalk genes were mainly involved in PI3K-Akt signaling pathway, MAPK signaling pathway, calcium signaling pathway, insulin signaling pathway and neurotrophin signaling pathway. Previous studies have found that enhancing the PI3K-Akt signaling pathway in the central nervous system can improve memory function *in vivo* in mouse AD models and human trials [31]. MAPK signaling cascades play a role in mediating the AD-related pathological effects of apoE4 in the hippocampus [32]. The above results indicate that the crosstalk genes on the pathways also play a crucial role in ADs occurrence and pathological progression.

### Molecular biology analysis of autophagy-related prognostic genes

Multivariate Cox regression analysis was performed to construct a prognostic gene model based on two prognostic autophagy-related crosstalk genes (CD40 and SMAD7), which could predict the overall survival of AD patients with medium-to-high accuracy. The CD40 receptor is a member of the tumor necrosis factor superfamily of transmembrane receptors. A previous study found that the pathological features (such as amyloid burden, astrocytosis and microgliosis that are typical of AD-like pathology in these transgenic mouse strains) are reduced in mouse models deficient for CD40 compared with their littermates where CD40 is present [42]. In addition, the pattern of expression of CD40 has been reported to be altered in the brains of AD patients as well as in several animal models of AD [43].

In this study, the expression level of CD40 in AD patients was higher than that in controls, which indicated that high expression of CD40 was associated with AD pathological progression. It has been suggested to play a role in Aβ metabolism in AD [44]. Interaction of CD40 with its ligand CD40L mediates a broad range of immune and inflammatory responses in the periphery and in the central nervous system [45]. Innate immune and inflammatory responses play an important role in the accumulation and progression of amyloid in AD [46]. Dyad of CD40/CD40 ligand fosters neuroinflammation at the blood–brain barrier (BBB) and is regulated through JNK signaling [47]. The BBB plays a key role in the generation and maintenance of chronic inflammation during AD [48].

These combined evidence suggest that CD40 has a broad role in AD. Previous studies have reported that nuclear SMAD7 and TGF-beta1 levels were markedly upregulated in cortical brain regions of the TgCRND8 mice (a mouse model of familial AD) [49]. Moreover, TGF-beta1 may amplify Aβ (1-42) (accumulation of the Aβ peptide in the brain is a crucial factor in the development of AD)-mediated neurodegeneration in AD through SMAD7 and beta-catenin interaction and nuclear localization. Another study showed that inhibiting cellular SMAD7 levels significantly ameliorated the Aβ (1-42)-mediated suppression of TGF-beta1-inducible transcription reporter activity, whereas SMAD7 transfection downregulated TGF-beta1-inducible transcription reporter activity [50]. Our results revealed that the expression level of SMAD7 was higher in AD patients than in controls, suggesting that inhibition of SMAD7 may be beneficial for AD.

Our study has some limitations. *In vivo* and *in vitro* experiments should be performed to further confirm our results. In summary, we performed comprehensive bioinformatics analysis and identified the autophagy-related prognostic gene signature containing two genes (CD40 and SMAD7) for AD patients.

## CONCLUSION

AD is a multifactor disease involving multiple signaling pathways, and the current methods for exploring new therapeutic targets need to be further enriched. Therefore, considering the core role of autophagy in AD and its interaction with other signaling pathways, this article provides a new method for mining autophagy-related biomarkers in AD. The molecular mechanism of autophagy in AD has not been explored from the perspective of pathway crosstalk. This article used the DESeq2 package to screen out differentially expressed lncRNAs.

The PAGI algorithm was used to explore the pathways related to AD, and the crosstalk pathways related to autophagy were screened through the number of autophagy genes in the pathways and literature. The mRNAs on the crosstalk pathways related to autophagy were reserved as feature mRNAs. The WGCNA algorithm was used to extract the coexpression module of feature mRNAs and lncRNAs. Next, we applied clinical data to the genes in the coexpression module to obtain prognostic genes. Finally, CD40 and SMAD7 were identified as prognostic genes in AD. Based on the external AD dataset and literature, the role of the extracted prognostic genes in AD was confirmed. However, the exact mechanism underlying how these genes affected the prognosis of AD should be verified by more accurate experiments.

## Figures and Tables

**SUPPLEMENTAL TABLE 1:**
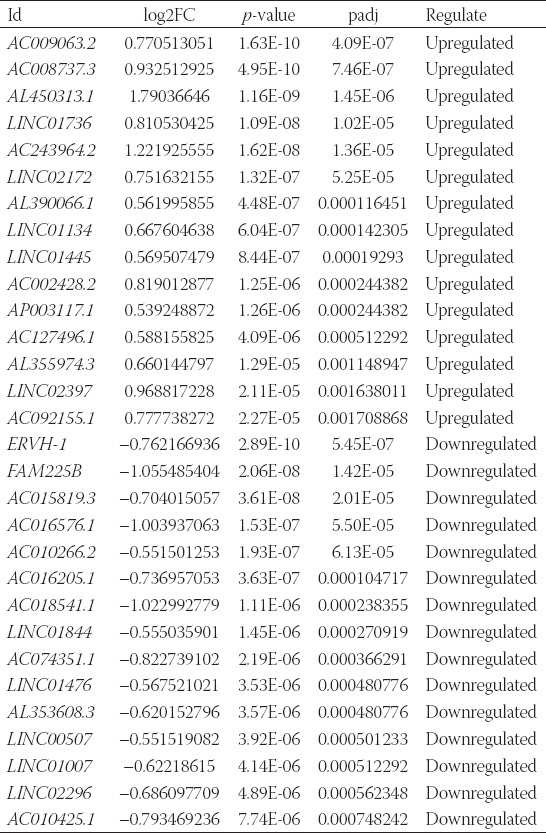
Top 30 differential genes

**SUPPLEMENTAL TABLE 2:**
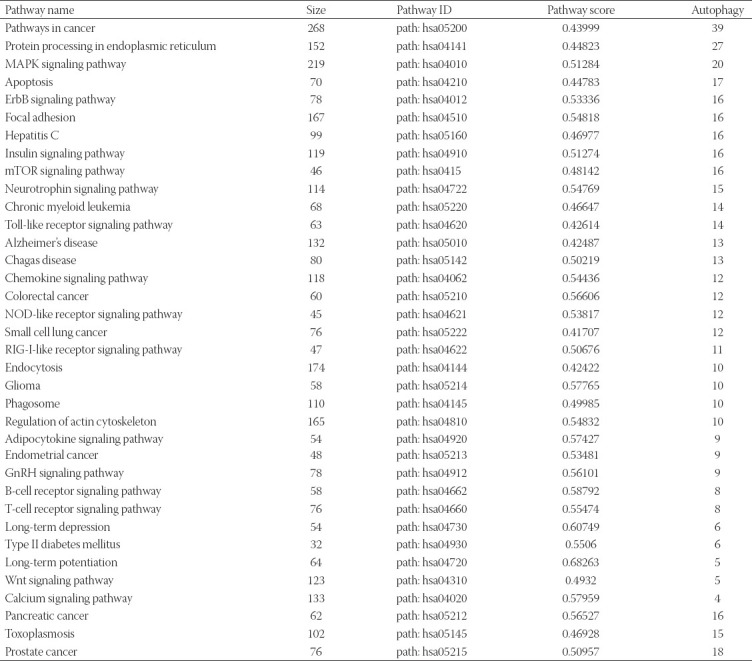
Thirty-six autophagy-related pathways

**SUPPLEMENTAL TABLE 3:**
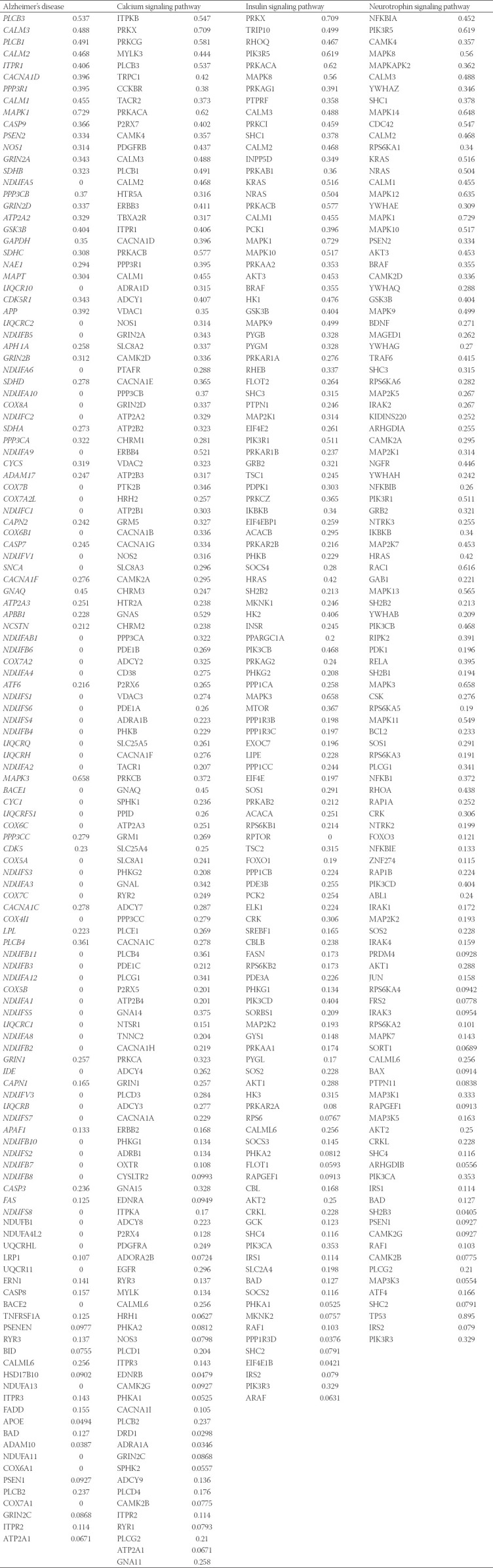
Crosstalk genes and their crosstalk scores on 10 autophagy-related crosstalk pathways

**SUPPLEMENTAL TABLE 4:**
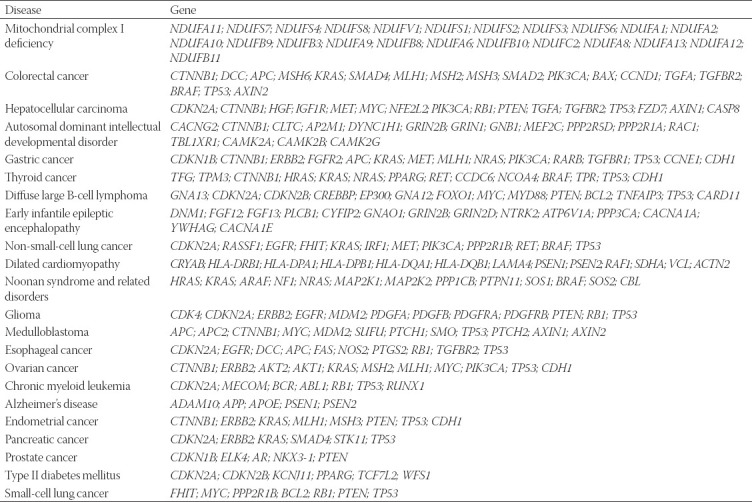
Genes associated with other diseases

**SUPPLEMENTAL TABLE 5:**
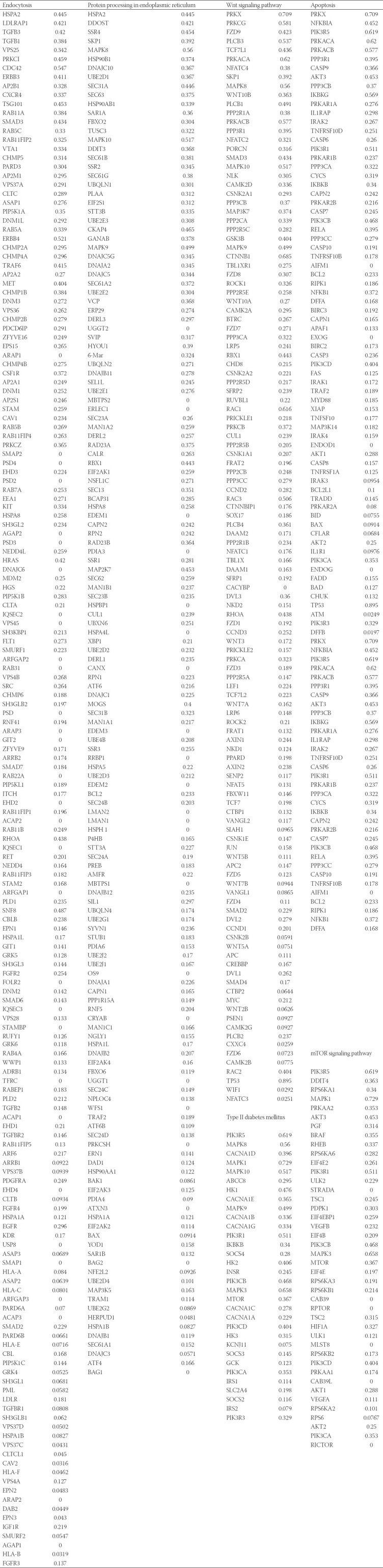
Definition of survival time and survival status of AD patients. Because the age of patients over 90 years old in the clinical data is shown as 90+, we set the observation starting point as the time of diagnosis of each patient and the observation ending point as 90 years old. The survival status of patients with a death age of 90 + is alive, while the survival status of patients with a death age of less than 90 is dead, and the survival time of 82 patients was finally obtained. The clinical data downloaded from the AMP-AD database were sorted and screened, and then, the survival time and clinical characteristics (braaksc, ceradsc, and dcfdx_lv: Clinical cognitive diagnosis summary at last visit) of 82 patients were obtained
